# Supplementary material for: Dietary Sources of Fructose and Its Association with Fatty Liver in Mexican Young Adults
Source: Nutrients. 2019 Feb 28;11(3):522. doi: 10.3390/nu11030522 (PMC6470703; doi:10.3390/nu11030522)
Supplement: Supplementary file 1 [file nutrients-11-00522-s001.zip › Supp_Figure 1.docx]

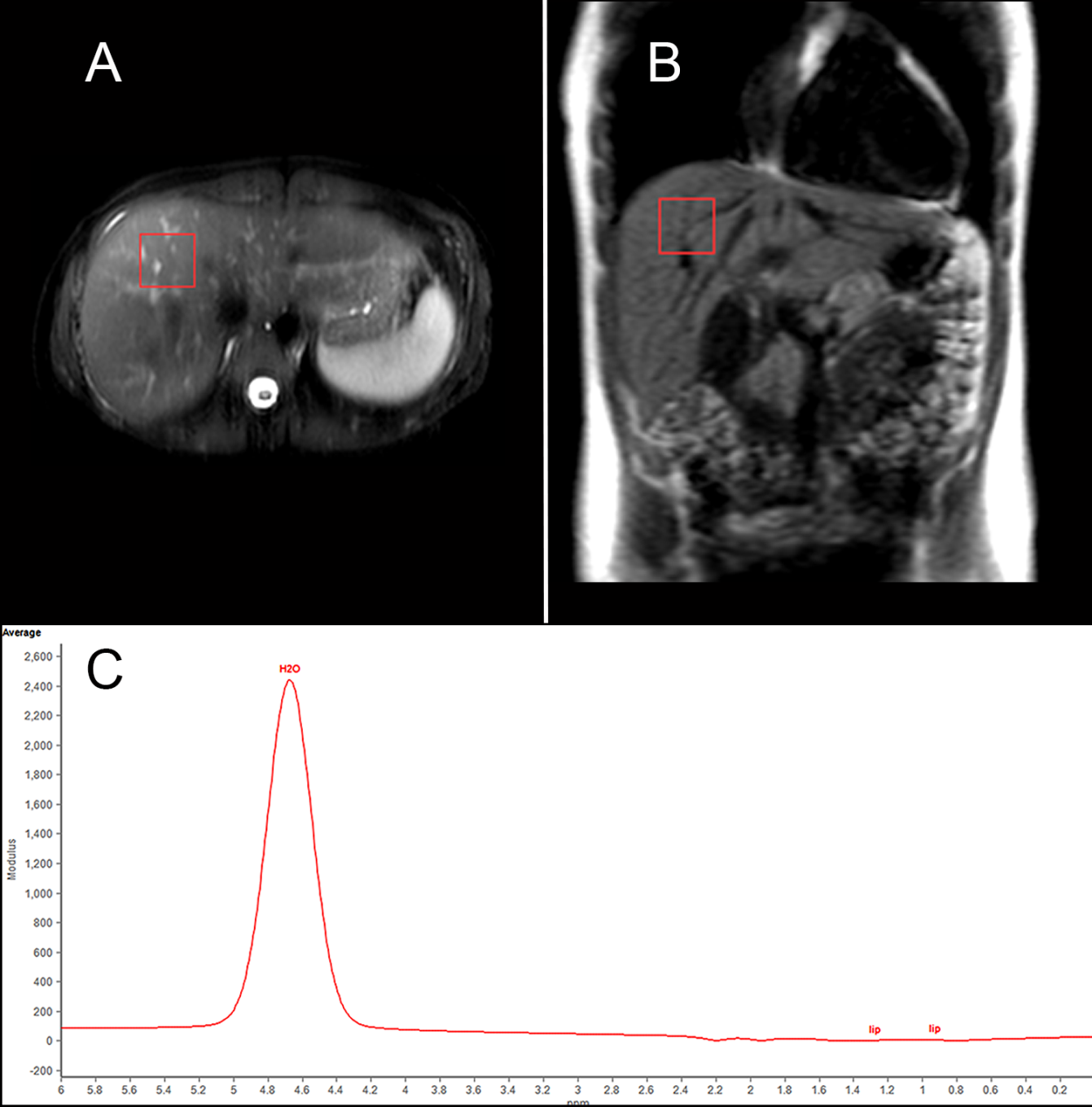


Figure 1. Acquired images and spectra for fat fraction quantification. A and B, T2-weighted images in axial and coronal planes showing voxel location in the right lobe of the liver. C, H1 magnetic resonance spectrum depicts the resonances used for calculation of the triglycerides content: water (peak at 4.7 ppm), methylene (peak at 1.3 ppm, [CH2]n) and methyl (peak at 0.9 ppm, CH3).
